# A pre-specified surgical-complexity index is associated with a graded increase in periprosthetic joint infection after total knee arthroplasty: a competing-risks analysis using coronal alignment as a negative control

**DOI:** 10.5194/jbji-11-705-2026

**Published:** 2026-11-11

**Authors:** Wayne Hoskins, Charles Gusho, Kenrick Rosser, Matthew Lim, Kate Dunstall, Kelly G. Vince

**Affiliations:** 1 Northland Orthopaedic Research Consortium, Whangārei Hospital, Te Whatu Ora Te Tai Tokerau, Whangārei, Aotearoa, New Zealand; 2 Faculty of Medicine, Dentistry and Health Sciences, The University of Melbourne, Parkville, Victoria, Australia; 3 Northland Knee & Hip, Whangārei, Aotearoa, New Zealand; 4 Missouri Orthopaedic Institute, University of Missouri, Columbia, Missouri, USA

## Abstract

Prediction of periprosthetic joint infection (PJI) after total knee arthroplasty (TKA) relies almost wholly on host factors, and whether the operation itself carries infection risk independent of the patient is unknown. We analyzed 2920 primary TKAs in 2350 patients from a single institution with national joint registry linkage, using death as a competing risk and coronal alignment as a negative-control exposure. A pre-specified surgical-complexity index counted five construct markers at the index operation: constraint, a tibial stem, a femoral stem, a previous implant and a pre-existing extra-articular deformity or fracture. Septic revision was the primary outcome and aseptic revision the control. Fine–Gray subdistribution hazard models with patient-level clustering estimated complexity and, in the paired-radiograph subset, neutral-alignment effects on both outcomes. There were 51 septic and 104 aseptic revisions and 525 deaths over a median follow-up of 7.4 years. Each additional complexity marker raised the subdistribution hazard of PJI (sHR 1.61, 95% CI 1.27–2.04) but not of aseptic revision (sHR 1.06, 95% CI 0.84–1.33); 5-year PJI incidence rose from 1.5% with zero or one marker to 5.9% with two or more. Neutral coronal alignment was protective against aseptic revision (sHR 0.42, 95% CI 0.26–0.67) but null for PJI (sHR 0.87, 95% CI 0.41–1.87). Adjustment for skin-to-skin operative time, available for 93% of the cohort, moved the complexity estimate in the same knees only from 1.58 to 1.55 and attributed 5 % of the effect to operative time. These are observational associations and not causal effects, but surgical exposure merits consideration alongside host factors in infection-risk assessment.

## 1 Introduction

Predictive models for periprosthetic joint infection (PJI) after total knee arthroplasty (TKA) typically ask which patient is at risk (Bagheri et al., 2025; Kotzur et al., 2024; Li et al., 2026; Lucenti et al., 2024; Maradit Kremers et al., 2025; Schiffner et al., 2020), yet few studies question the operation itself. A PJI is the most morbid and costly failure mode after TKA (Premkumar et al., 2021), and its risk factors and risk stratification have generated an expanding literature (Chong et al., 2025; Kotzur et al., 2024; Maradit Kremers et al., 2025). Prediction matters because prevention can only be directed at the patients that a model identifies, and a risk that is not predicted is one the surgeon cannot act on.

Prediction models share large numbers of demographic, comorbidity and laboratory variables, and are trained on large cohorts with relatively few PJIs (Li et al., 2026; Maradit Kremers et al., 2025). All place the patient, rather than the operation, at the center of risk, and the operation itself has attracted far less study; robotic and navigated assistance, for instance, does not change PJI risk in matched cohorts (LaValva et al., 2024). The operative factors that have been linked to infection are largely generic surrogates of surgical burden, such as prolonged operative time, blood transfusion and prolonged length of stay (Bagheri et al., 2025; Lucenti et al., 2024), rather than the specific complexity of the surgery. Operative time is the best established of these surrogates and is biologically coherent, but it is known only once the operation is over and cannot inform consent, prophylaxis or the planning of a list. Two preoperative calculators have been described, using six and 17 variables respectively (Berbari et al., 2012; Tan et al., 2018). Neither counts the mechanical demands of the operation, although a previous open surgical procedure was the most influential of the 42 factors screened in the larger of them. What has not been asked is whether the mechanical demands of the operation, the deformity to be corrected and the construct required, relate to PJI. Coronal alignment, meanwhile, has been studied almost entirely for mechanical failure and hardly at all for infection (Hoskins and Gusho, 2026).

## 2 Methods

This retrospective cohort study analyzed prospectively collected data on primary TKAs performed at a single institution between 2010 and 2024, cross-linked to a national joint registry to capture revisions performed elsewhere. All patients had at least 2 years of potential follow-up. The analytic cohort comprised 2920 primary TKAs in 2350 patients with long-leg radiographic assessment and complete operative records. Bilateral procedures were retained and handled statistically by patient-level clustering, and the study was approved by the regional ethics committee.

The primary outcome was septic revision (any revision for a diagnosis of PJI) and the control outcome aseptic revision (any revision for a non-PJI cause). Death without revision was treated as a competing event, and each outcome treated the other revision type as competing. The primary exposure was a pre-specified surgical-complexity index – the count of five construct markers present at the index operation. These were constraint (condylar-constrained or hinge), a tibial stem, a femoral stem, a previous surgical implant and a pre-existing extra-articular deformity or prior fracture. The five were fixed before any analysis against three criteria stated in advance: each is recorded objectively rather than by judgment; each is knowable from the preoperative radiograph and the operative plan; and each is a case-complexity field not captured by national registries (Hoskins et al., 2023). None was selected on the basis of its association with infection, and the index was left unweighted because weighting rare markers on 51 events would fit noise.

Every constrained implant was stemmed, so constraint and stem fixation were collinear and could not be separated at the component level, which is why the aggregated index was pre-specified as the primary exposure. The index ranged from 0 to 5 and was analyzed as an ordinal dose-response and as its components. In the subset with paired preoperative and postoperative long-leg radiographs, coronal alignment was quantified as neutral mechanical alignment, defined as a postoperative mechanical hip-knee-ankle (mHKA) angle within 3 degrees of neutral, and was examined additionally as the continuous deviation from neutral and as the postoperative Kennedy zone of the weight-bearing line (Hoskins and Gusho, 2026). Skin-to-skin operative time was obtained from the national registry data linkage, giving operative time for 2722 knees (93 %); it was not pre-specified. Surgeon identity and the alignment target intended by the operating surgeon were not recorded.

### 2.1 Statistical analysis

Because a substantial proportion of patients died before revision, the Fine–Gray subdistribution hazard model was used so that the modeled quantity was cumulative incidence in the presence of the competing risk of death (Fine and Gray, 1999; Lacny et al., 2015). The index was entered as a continuous ordinal predictor adjusted for age, body mass index (BMI), American Society of Anesthesiologists (ASA) grade of at least 3 and sex; the identical model was fit for the control outcome and coronal alignment tested on both outcomes in the paired-radiograph subset. The control outcome follows the negative-control framework (Lipsitch et al., 2010). Firth's penalized-likelihood correction was pre-specified for component-level inference, because rare binary components were expected to separate (Firth, 1993; Heinze and Schemper, 2002). Five-year cumulative incidence was estimated by the Aalen–Johansen method. Robustness to unmeasured confounding was quantified with the E value, the minimum association a confounder would need with both exposure and outcome to explain an estimate away (VanderWeele and Ding, 2017). The primary model was also refit with operative time as a covariate, the fraction of the effect transmitted through it estimated by the difference method. Analyses were performed in Python (Python Software Foundation, Wilmington, DE, USA) and R (R Foundation for Statistical Computing, Vienna, Austria), with P<0.05 considered statistically significant.

## 3 Results

### 3.1 Cohort

The cohort comprised 2920 primary total knee arthroplasties in 2350 patients. The mean age was 69.4 years, 52 % were men, the mean BMI was 31.7 kg m^−2^, 29 % had an ASA grade of at least 3 and 16 % identified as New Zealand Māori. Median follow-up was 7.4 years (interquartile range 4.4 to 10.7). There were 51 septic revisions, 104 aseptic revisions and 525 deaths, and the overall 5-year cumulative incidence of PJI was 1.7 %. The 525 deaths are 18.0 % of the cohort over a median of 7.4 years, lower than the 27.8 % reported at the same median follow-up in 98 778 primary knees in Scotland (Yapp et al., 2022) and the reason a competing-risks framework was necessary. Baseline characteristics are shown in Table 1; complexity markers were present in 525 knees (18.0 %) and 177 (6.1 %) carried two or more.

**Table 1:** Baseline characteristics by surgical-complexity stratum.

Characteristic	0 markers	1 marker	≥2 markers
	( n=2395 )	( n=348 )	( n=177 )
Age, mean (yr)	69.7	68.1	68.3
BMI, mean	31.5	32.7	32.7
Male, %	54	46	29
ASA ≥ 3, %	28	38	34
Septic revisions, n	35	6	10
Aseptic revisions, n	86	8	10

### 3.2 Surgical complexity and infection

Each additional complexity marker raised the subdistribution hazard ratio (sHR) of PJI (sHR 1.61, 95 % confidence interval [CI] 1.27 to 2.04, P<0.001 ), with a model concordance of 0.68 (Table 2, Fig. 1). The association was independent of the measured host factors: male sex was associated with infection (sHR 2.83, 95 % CI 1.49 to 5.39), whereas ASA of at least 3 and body mass index pointed the same way without reaching significance at 51 events (Table 2). Five-year PJI incidence was 1.5 % at zero markers (95 % CI 1.1 to 2.1), 1.5 % at one marker (95 % CI 0.6 to 3.5) and 5.9 % at two or more (95 % CI 3.2 to 10.8), indicating a practical threshold at two markers (Table 3, Fig. 2A). The E value was 2.60, and 1.86 for the confidence limit nearer the null, so an unmeasured confounder would need an association of at least 2.60 with both the index and PJI, beyond age, sex, BMI and ASA, to explain it away.

**Figure 1:**
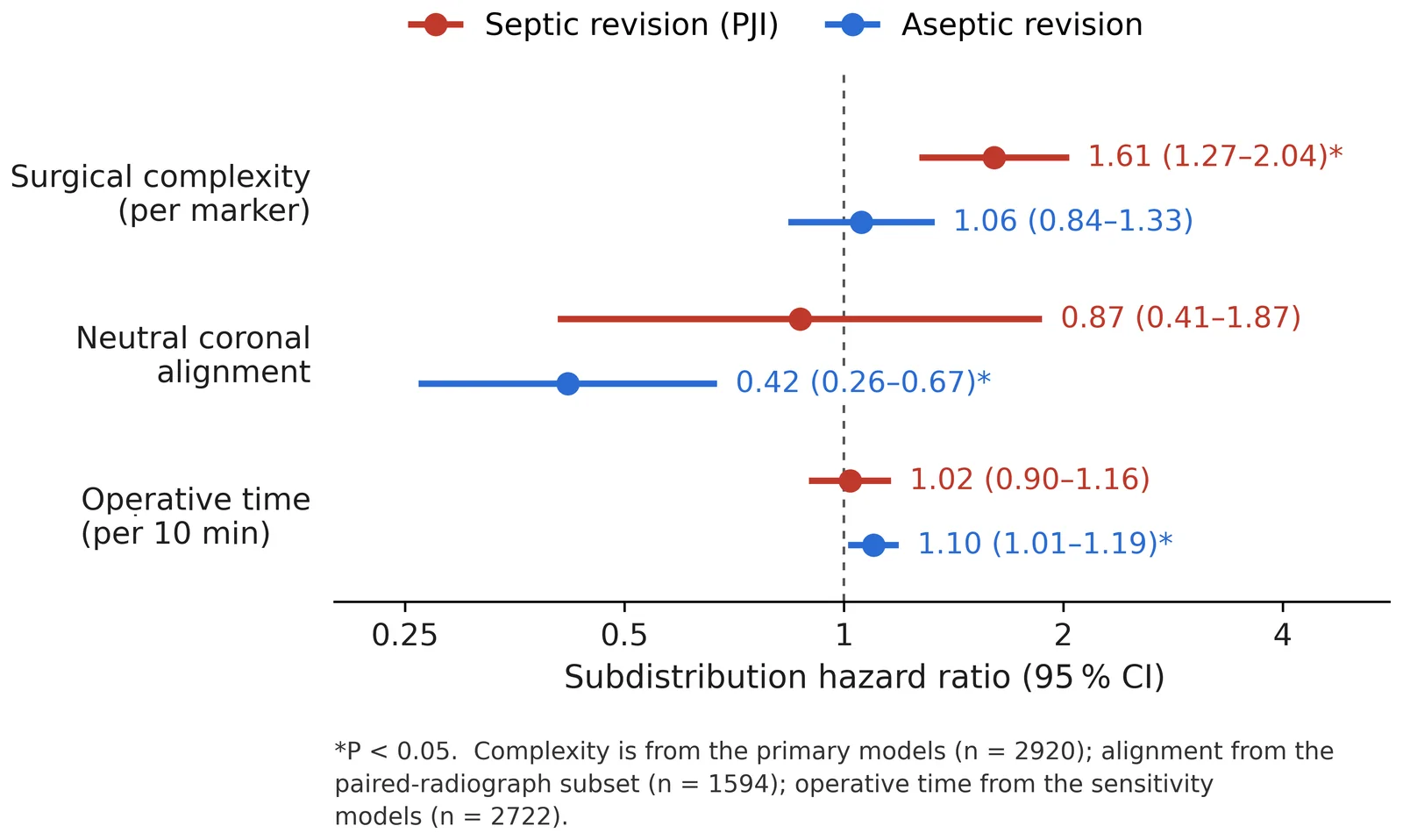
Double dissociation of surgical complexity, coronal alignment and operative time. Subdistribution hazard ratios with 95 % confidence intervals for each exposure against each failure mode, with death treated as a competing risk.

**Figure 2:**
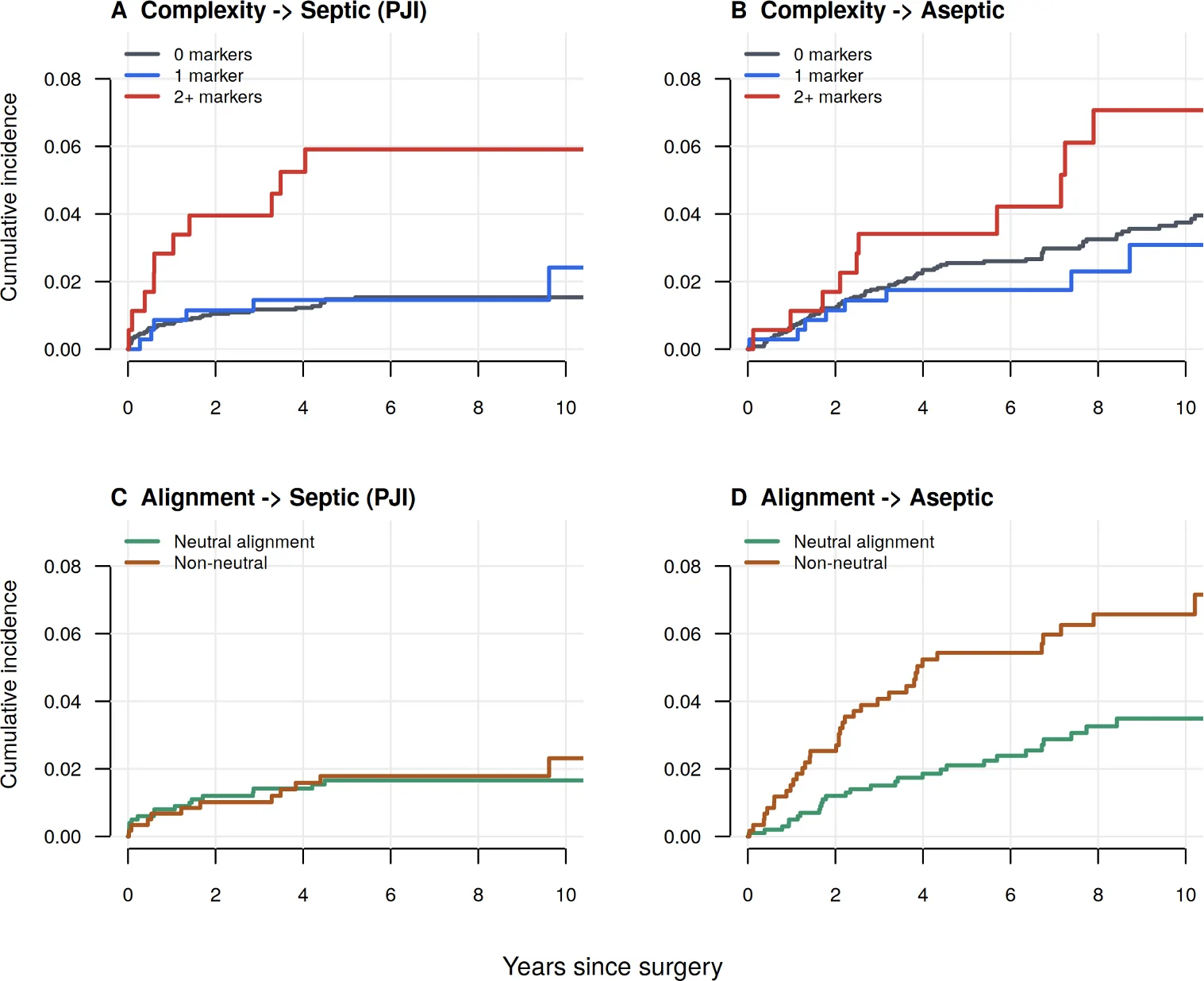
Cumulative incidence of septic and aseptic revision by surgical complexity **(A, B)** and by neutral coronal alignment **(C, D)**.

**Table 2:** Fine–Gray models for periprosthetic joint infection (death competing).

Variable	sHR	95 % CI	P
Complexity index (per marker)	1.61	1.27–2.04	<0.001
Male sex	2.83	1.49–5.39	0.002
ASA ≥ 3	1.38	0.69–2.74	0.36
BMI (per 5 units)	1.18	0.88–1.58	0.28
Age (per decade)	1.03	0.75–1.41	0.87

**Table 3:** Five-year cumulative incidence of PJI by complexity stratum.

Complexity	N	PJI	5-year
markers		events	CIF
0	2395	35	1.5 %
1	348	6	1.5 %
≥2	177	10	5.9 %

Operative time was recorded for 2722 knees (93 %), with a median of 85 min (interquartile range 72 to 100), and rose with the complexity index from a mean of 85 min at no markers to 111 at three and 122 at four, although not strictly monotonically across the sparsely populated middle strata. In the 2722 knees with operative time, adding it to the primary model moved the complexity-PJI subdistribution hazard ratio from 1.58 to 1.55 (95 % CI 1.21 to 1.98, P<0.001 ); operative time itself was not associated with septic revision (sHR 1.002 per minute, 95 % CI 0.990 to 1.015, P=0.72 ), and a difference-method decomposition attributed 5 % of the complexity effect to operative time. In the aseptic control, the pattern reversed, operative time being associated with revision (sHR 1.009 per minute, 95 % CI 1.001 to 1.017, P=0.023 ) but not with the complexity index (sHR 0.98, 95 % CI 0.74 to 1.29, P=0.87 ) – a second dissociation (Figs. 1 and 3).

**Figure 3:**
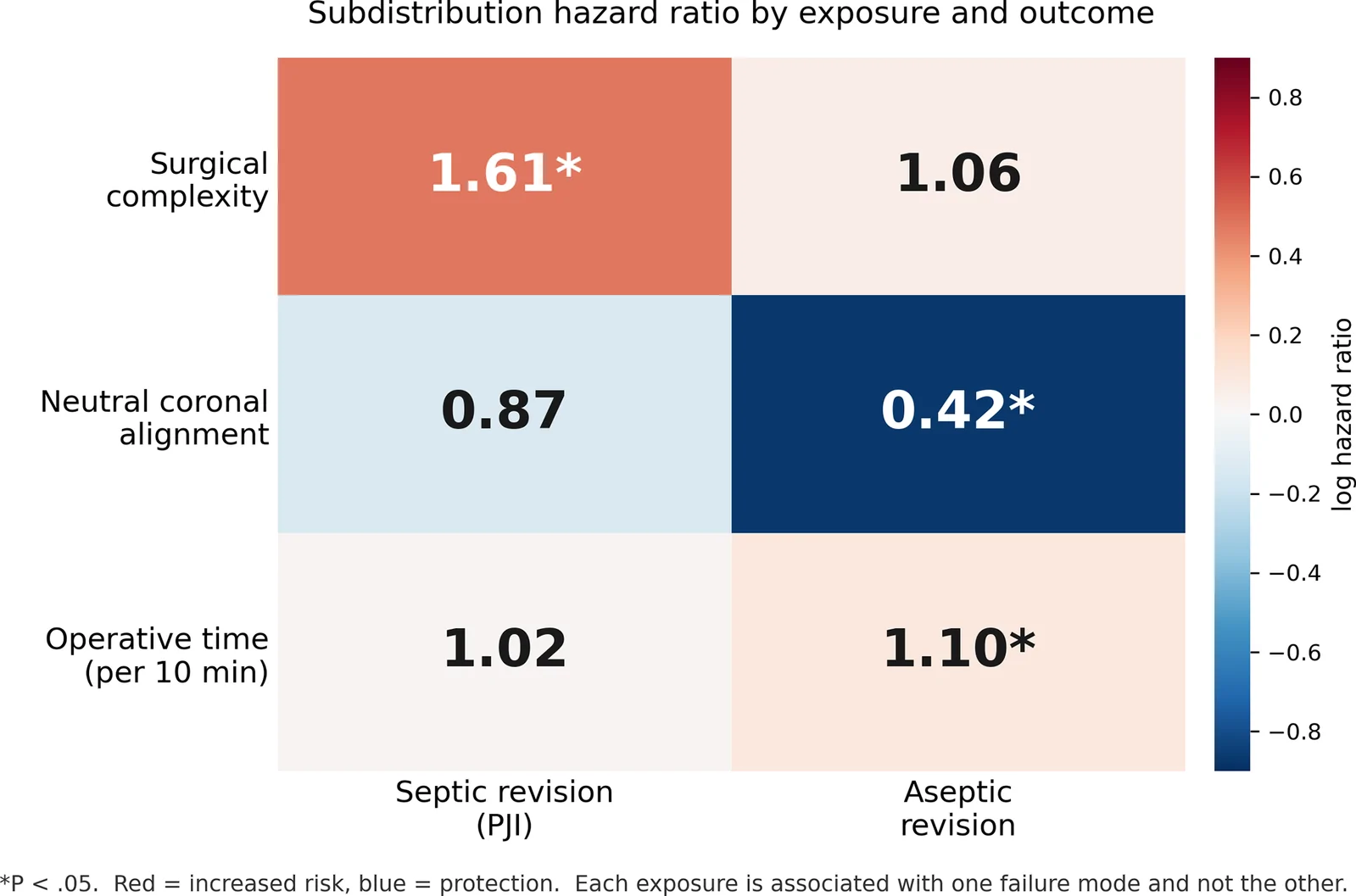
Dissociation matrix. Subdistribution hazard ratio by exposure and outcome; each exposure is associated with one failure mode and not the other.

### 3.3 The negative-control dissociation

The identical complexity index was not associated with aseptic revision (sHR 1.06, 95 % CI 0.84 to 1.33, P=0.64 ). In the paired-radiograph subset of 1594 knees, neutral coronal alignment was protective against aseptic revision (sHR 0.42, 95 % CI 0.26 to 0.67, P<0.001 ) but unrelated to PJI (sHR 0.87, 95 % CI 0.41 to 1.87, P=0.72 ). Each degree of deviation from neutral raised aseptic revision (sHR 1.22 per degree, 95 % CI 1.12 to 1.33) but not PJI (sHR 1.08, 95 % CI 0.94 to 1.23), and a central Kennedy zone showed the same pattern (aseptic sHR 0.38, 95 % CI 0.22 to 0.63; PJI sHR 0.60, 95 % CI 0.24 to 1.48). Surgical complexity and coronal alignment therefore showed opposite patterns across the two failure modes, a double dissociation (Figs. 1–3).

### 3.4 Component-level estimation

Fitting the five markers individually produced gross separation, the constraint coefficient inflating without bound because rare markers perfectly predicted infection within small cells, confirming that component-level inference requires the pre-specified penalized-likelihood estimator and supporting the aggregated index as the primary analysis.

## 4 Discussion

This study tested whether the operation itself carries PJI risk, indexed by a pre-specified count of complexity markers, with coronal alignment as a negative control and death as a competing risk. Surgical complexity was associated with a graded increase in infection, independent of the measured host factors, whereas coronal alignment was not, and 5-year infection incidence rose roughly four-fold once two or more markers were present. Alignment reproduced its established protection against aseptic revision but was unrelated to infection. These are associations in a single retrospective cohort and are hypothesis generating rather than causal. The practical implication we would draw is a modest one: that the more a knee demands of the operation, the more it may demand of infection prevention.

Male sex, higher ASA and obesity are well-established infection risks (Bagheri et al., 2025; Li et al., 2026; Lucenti et al., 2024); male sex reproduced clearly here, whereas ASA and BMI pointed the same way without reaching significance. What is added is a surgical-exposure axis that host-factor models and national registries do not capture: registries record who was operated on but not how demanding the operation was; they undercount infection unless augmented with microbiology or chart review (Anneberg et al., 2023, 2024; Koh et al., 2017). Case complexity, previous operations, bone defects and preoperative alignment are seldom recorded on their forms (Hoskins et al., 2023). That robotic and navigated assistance do not change PJI risk is consistent with this reading, because those technologies change how a target is reached rather than the size of the surgical task (LaValva et al., 2024).

Operative time deserves specific comment, because the interval over which a wound is open is a well-supported determinant of infection (Chong et al., 2025; DeMik et al., 2022; Pacha et al., 2025) and might be thought to confound this association. Constraint, stemmed fixation, the removal of a previous implant and the correction of an extra-articular deformity all lengthen the operation, so operative time lies on the causal path from complexity to infection rather than behind both, and conditioning on it estimates only that part of the effect that does not run through prolonged exposure (Schisterman et al., 2009). When we nonetheless conditioned on it, the association was barely disturbed and only 5 % of the effect traveled through duration. We do not claim that the complexity count predicts infection better than operative time but that it is available earlier – from the radiograph and the operative plan rather than from the completed operation.

**Figure 4:**
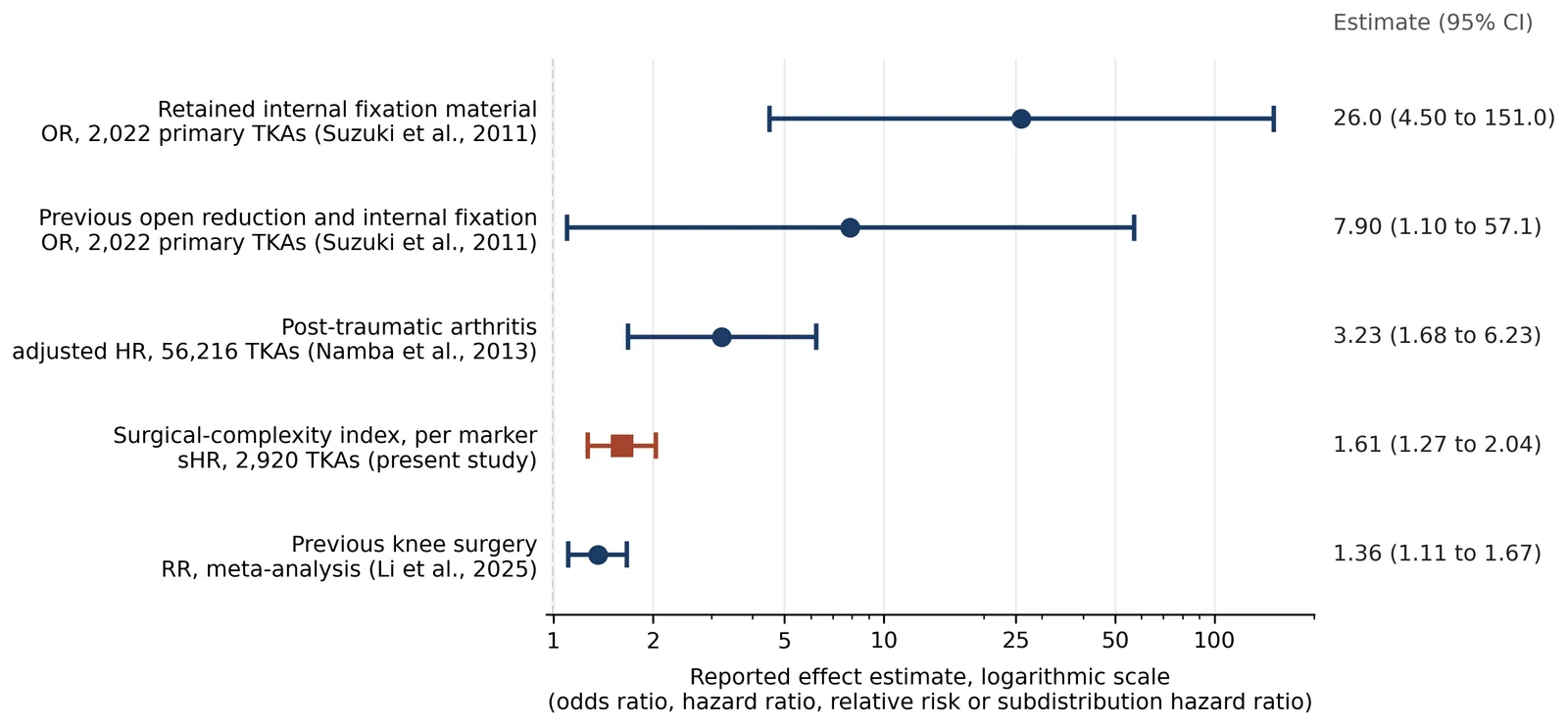
Reported associations between markers of previous surgery or retained material and infection after knee replacement in two independent cohorts and one meta-analysis, with the per-marker estimate from the present study. Estimates are odds ratios, hazard ratios, relative risks and subdistribution hazard ratios from different designs and endpoints, shown on a common logarithmic scale and not pooled.

Two external series bear on the same chain. Costa et al. (2020) matched 74 primary knees receiving a varus-valgus constrained implant one to one with 74 posterior-stabilized knees and found 5-year survivorship free of revision for infection of 91.9 % against 100 % ( P=0.013 ), whereas survivorship free of revision for every other cause did not differ. This is the same pattern, septic but not aseptic, found by a different design. Their groups differed by 7.1 min of mean operative time against a contrast of six infections and none, and in our cohort the equivalent difference was 6.3 min. In the largest series of this outcome – 56 216 primary knees with 404 deep infections – operative time was associated with infection on univariable analysis but did not enter the final adjusted model, and those authors read it as a proxy for the complexity of the procedure (Namba et al., 2013).

A multifactorial mechanism is available for the association of surgical complexity with PJI. In the established animal model, a foreign body reduced the inoculum of *Staphylococcus aureus* needed to establish infection by orders of magnitude, and granulocytes at an implant surface killed poorly despite opsonization (Zimmerli et al., 1982, 2004). A constrained, stemmed construct presents a larger foreign surface than a primary condylar implant, and the knees this index counts often carry retained material already: sonication of osteosynthetic implants from asymptomatic patients grew bacteria in 27 %, seven times more often at the knee than at the hip (Fuchs et al., 2019). Previous knee surgery and a thicker soft-tissue envelope independently raise infection risk after replacement (Li et al., 2025; Vahedi et al., 2020). Three further cohorts support the construct (Fig. 4): previous internal fixation, and more strongly retained material, were independent risk factors in a cohort of 2022 knees (Suzuki et al., 2011); post-traumatic arthritis carried an adjusted hazard ratio of 3.23 in 56 216 knees (Namba et al., 2013); and of 42 factors screened in 27 717 patients, the most influential was a previous open surgical procedure (Tan et al., 2018).

These results sit alongside the two published calculators rather than replacing them (Table 4). Both discriminate better than the model reported here, from six and from 17 variables (Berbari et al., 2012; Tan et al., 2018). What is offered is not discrimination but an axis neither calculator counts and a decomposition showing that most of what it carries does not travel through the duration of the operation.

**Table 4:** Preoperative prediction of periprosthetic joint infection: the two published models and the present study.

	Berbari et al. (2012)	Tan et al. (2018)	Present study
Design	Matched case-control	Single-center cohort with external validation	Registry-linked cohort, death as competing risk
Patients	339 cases, 339 controls	27 717	2350 (2920 knees)
Infections	339	1035	51
Variables in the model	6	17	5-marker index plus four host covariates
Discrimination	C-statistic 0.722	AUC 0.83 (0.84 external)	C-statistic 0.68
Operative time	In the model	Excluded by design	Sensitivity analysis
Surgical-complexity axis	No	Prior open procedure highest weighted	Pre-specified index

No single marker accounted for the association. The markers were uncommon and highly correlated, every constrained knee was also stemmed and the component model separated, so the signal came from the graded accumulation of complexity rather than from any one component. The negative-control result is the methodological core of the study. If coronal alignment reduced aseptic revision only through residual confounding, it should also have tracked infection, and it did not, which is more consistent with two pathways than with a shared bias (Lipsitch et al., 2010). The argument does not require the alignment-aseptic association to be free of confounding but the opposite, because confounders acting across failure modes should have moved the alignment-infection estimate as well. What it cannot exclude is a confounder specific to aseptic failure, and a target alignment differing between surgeons is exactly such a candidate. Neither surgeon identity nor the intended alignment strategy was recorded, so the alignment findings should be read as reproducing an established association rather than as a comparison of alignment philosophies.

For the surgeon, the infection conversation should include the operation and not only the patient. A knee needing added constraint, stems or removal of a previous implant, or carrying deformity from a previous fracture represents a larger exposure and a compromised envelope, and the four-fold rise at two or more markers marks the cases in which intensified prophylaxis and shared decision-making are warranted. The index is not a validated calculator.

This study has limitations. It is retrospective and from a single institution, the estimates are associations rather than causal effects and specific values should be regarded as internally valid only. With 51 infections the analysis supports a small pre-specified exposure set and a directional negative-control test rather than an individualized predictor, and its concordance of 0.68 is below that of both published calculators. Constraint and stems partly mark revision-prone knees, so confounding by indication cannot be excluded. The alignment-infection null rests on 27 infections and is consistent with, but not proof of, the absence of an effect. Operative time was available for only 93 % of the cohort; the 198 knees without it carried proportionally more aseptic revisions than the cohort, and a decomposition on 47 septic events is imprecise. The null association between operative time and septic revision here is not evidence that duration is unimportant to infection in general, and larger series using a broader surgical site infection endpoint do report one (Namba et al., 2013). The E value bounds how strong an unmeasured confounder would have to be but does not exclude one. Finally, the index aggregates heterogeneous markers whose component-level effects require the penalized-likelihood estimator noted above.

## 5 Conclusions

Surgical complexity was associated with a graded increase in PJI after primary TKA, independent of the measured host factors, whereas coronal alignment was not, and the two exposures showed an opposite pattern across septic and aseptic failure. Adjustment for operative time left that association essentially intact and attributed only a small fraction of it to duration. Surgical exposure deserves a place alongside host factors in how infection risk is understood, and external validation in a cohort with operative time recorded is the necessary next step.

## Data Availability

The data that support the findings of this study are available from the corresponding author on reasonable request. The data are not publicly available because they contain information that could compromise the privacy of research participants and are held under the governance of the linked national joint registry.

## Code availability

The analysis code (R and Python) that supports the findings of this study is available from the corresponding author on reasonable request.
